# Implementation of the Hospitalist System and In‐Hospital Mortality Among Patients With Cancer: Using the National Health Insurance Cohort Data

**DOI:** 10.1002/cam4.71207

**Published:** 2025-10-02

**Authors:** Yun Seo Jang, Il Yun, Yu Shin Park, Eun‐Cheol Park, Jaeyong Shin

**Affiliations:** ^1^ Department of Public Health Graduate School, Yonsei University Seoul Republic of Korea; ^2^ Korea Medical Institute Seoul Republic of Korea; ^3^ Institute of Health Services Research, Yonsei University Seoul Republic of Korea; ^4^ Department of Preventive Medicine Gachon University College of Medicine Incheon Republic of Korea; ^5^ Mo‐Im Kim Nursing Research Institute, Yonsei University College of Nursing Seoul Republic of Korea; ^6^ Department of Preventive Medicine Yonsei University College of Medicine Seoul Republic of Korea; ^7^ Department of Policy Analysis and Management College of Human Ecology, Cornell University Ithaca New York USA

**Keywords:** cancer, hospitalist, in‐hospital mortality, quality of healthcare

## Abstract

**Introduction:**

Hospitalists are directly responsible for inpatients, from hospitalization to discharge. Recently, Korea has started reimbursing hospitalist inpatient services. However, evidence of hospitalists being associated with improved healthcare quality is lacking. We investigated the association between the hospitalist system and reduced in‐hospital mortality among patients with cancer.

**Methods:**

This national population‐based retrospective cohort study included 398,732 patients with cancer from tertiary and general hospitals with hospitalists whose data were extracted from the Korean National Health Insurance Service Cohort Database in 2021. In‐hospital mortality data was obtained, defined as the presence of a record of death between admission and discharge. To increase comparability, we performed a 1:3 propensity score matching based on sex, age, hospital type, hospital region, Charlson Comorbidity Index (CCI), and primary cancer type. We used generalized estimation equation models to estimate the adjusted odds ratios (OR) for in‐hospital mortality.

**Results:**

Patients under the hospitalist system had a lower risk of in‐hospital mortality (OR: 0.91; 95% CI: 0.87–0.96). Specifically, patients in their 80s (OR = 0.31; 95% confidence interval [CI]: 0.19–0.52) and those with high CCI (OR = 0.93; 95% CI: 0.87–0.99) had a more significant association with lower in‐hospital mortality.

**Conclusions:**

Hospitalist services are associated with reduced in‐hospital mortality rates in cancer patients, which may be influenced by continuous patient management and expertise. Our results highlight the need for dedicated personnel stationed in hospital wards for improved outcomes of patients with cancer. Our results may encourage the government to consider the expansion of the current policies for efficient allocation of healthcare resources among hospitals in Korea.

## 1 Introduction

Globally, hospital systems are consistently operating to enhance patient safety management and increase efficiency [1, 2, 3, 4]. In Korea, where national health insurance is available, a low out‐of‐pocket burden has led to increased hospitalization rates [5]. Despite the significant number of hospital beds, there is a shortage of medical personnel for the management of inpatients. Consequently, the government introduced a policy of deploying hospitalists to address physician shortages in the wards. These hospitalists continuously reside in wards with inpatients, ensuring geographical accessibility and providing healthcare services exclusively for targeted patients. The policy began as a pilot project in 2016, targeting hospitals willing to participate, and later transitioned to an official program in January 2021 (Appendix S1).

Previous studies have reported that receiving services from hospitalists can reduce the length of stays [6], unnecessary hospitalizations [7, 8, 9, 10], and healthcare expenditures [8, 10, 11]. In Korea, positive outcomes, such as a decrease in hospital incidents and high patient satisfaction, have been observed through internal hospital evaluations during the pilot project phase [12, 13]. However, research on whether the program has led to an overall improvement in healthcare quality is limited. Furthermore, numerous previous studies evaluating the hospitalist system focused on specific patient groups, such as pediatric or older adult patients or those undergoing specific surgical procedures [14, 15, 16, 17, 18, 19]. However, to our knowledge, research evaluating whether hospitalists play a crucial role in managing critical diseases, particularly cancer, is scarce. In some hospitals, hospitalists work exclusively in the oncology wards [20, 21] for an effective acute hospital treatment tailored to individual cancer treatment plans [20], highlighting the importance of evaluating outcomes when providing a hospitalist system for patients with cancer [22, 23, 24].

Therefore, this study aimed to examine the in‐hospital mortality of hospitalized patients with cancer in Korea based on the presence of hospitalists. We hypothesized that cancer patients receiving care from hospitalists would experience a reduction in in‐hospital mortality, especially among those with multiple comorbidities, compared to those in general wards. Furthermore, we expected that hospitalist care would lead to improved healthcare quality, as evidenced by lower mortality rates, faster discharge times, and better coordination of care.

## 2 Methods

### 2.1 Data

This national population‐based retrospective cohort study utilized the National Health Insurance Service (NHIS) database in South Korea. The NHIS is a government‐managed social security system that provides universal healthcare coverage to all South Korean citizens, excluding those required to enroll in other mandatory medical support programs. The National Health Insurance Database provides claims data for academic research and policy development purposes [25]. It encompasses health‐screening records, medical utilization claims, sociodemographic characteristics, and mortality information for the entire South Korean population [25]. Among these, the claims data represent the most comprehensive dataset, covering the complete medical history of the entire population [25].

The study protocol was approved by the institutional review board at Yonsei University (Severance Hospital, IRB number = 4‐2023‐1230). The requirement for informed consent was waived because the data did not include personally identifiable information. This study adhered to the guidelines for reporting epidemiological observational studies on cohort research, thereby enhancing its transparency and rigor.

### 2.2 Study Population

We targeted patients with cancer in the NHIS cohort database using the codes “V027,” “V193,” and “V194.” [26] In Korea, the codes are specific designations under the National Health Insurance Act and the Special Treatment for Cancer Patients' Co‐payment Criteria, applying to cancer patients registered with the national system who have received treatment for 5 years under cancer‐specific ICD‐10 codes (C00–C97, D00–D09, D32–D33, D37–D48) [26]. This dataset was derived from claims data from 2017, and the follow‐up period was from January 2017 to December 2021, incorporating the most recent data available. Individuals included in this dataset were tracked and observed unless they were excluded due to death or emigration, in accordance with the National Health Insurance Act. As a result, the NHIS customized cohort data that we obtained included medical utilization records of all registered patients with cancer.

Because the inpatient hospitalist program was implemented in 2021, we only included data from that year for the extraction of relevant claims codes (*N* = 3,133,449). As hospitalists are deployed primarily in tertiary and general hospitals, we limited our study to patients admitted to these hospitals and excluded those who admitted to hospice wards to omit end‐stage cancer (*N* = 2,291,835). Cases in which the admission and discharge dates were the same (i.e., with a hospital stay of only 1 day) were defined as same‐day discharges and were excluded from the study. Therefore, the study population comprised 2,078,741 hospitalization claims, excluding cases with missing data (cases: 99,683, controls: 1,979,058).

After selecting the study population, we matched patients admitted to a specialized ward with those admitted to a general ward in a 1:3 ratio using propensity score matching (PSM), which is a statistical method used in observational studies to minimize bias. This approach prioritizes optimal matches first and identifies subsequent matches in hierarchical order until no further matches can be established (nearest‐neighbor matching). To calculate the probability of mortality, propensity scores were derived using logistic regression, considering covariates such as sex, age, hospital type, hospital region, and primary cancer type. Following the calculation of propensity scores, the OneToManyMTCH macro in SAS was employed to perform one to three greedy matches based on the propensity scores. Also, in Korea, unlike in some other countries, doctors (both hospitalists and non‐hospitalists) cannot personally designate patients, and there is no structure where a doctor is assigned to a specific patient. However, physicians can designate the wards where they reside or are in charge. This approach allowed us to indirectly assess the impact of hospitalist care based on the ward type, even though the direct assignment of patients to specific doctors is not possible in Korea. Therefore, the final study population comprised 398,732 inpatient claims (cases: 99,683, controls: 299,049).

### 2.3 Variables

The primary variable of interest was whether the patients received medical services from an inpatient hospitalist during their hospitalization. This was determined by extracting cases in which the inpatient hospitalist claim codes (AC201, AC202, and AC203) were billed to the patients' medical claim code variables. The dependent variable was in‐hospital mortality, defined as the presence of a record of death between admission and discharge dates.

We considered covariates related to patient and hospital characteristics. Patient characteristics included sex (male or female), age (< 30, 30–39, 40–49, 50–59, 60–69, 70–79, or ≥ 80 years), patient region (metropolitan, urban, rural), income level (quartile 1 [low] to 4 [high]), primary cancer type (lung, liver, colorectal, gastric, pancreatic, gallbladder/bile duct, breast, prostate, non‐Hodgkin lymphoma, leukemia, or others), and Charlson Comorbidity Index (CCI; 0, 1, or > 2). CCI, a measure of patient severity based on comorbidities, was determined using ICD‐10 codes for diagnoses, and weighted scores were assigned to 19 comorbid conditions based on their relative 1‐year mortality risk [27, 28]. We additionally adjusted for hospital type (tertiary general hospital or general hospital), hospital region (metropolitan, urban, or rural), and number of beds (quartile 1 [low] to 4 [high]).

### 2.4 Statistical Analysis

Descriptive statistics for in‐hospital mortality were presented as frequency (*N*) and percentage (%). The Chi‐squared test was performed to analyze and compare the overall characteristics of the study population. A generalized estimation equation was applied to evaluate repeatedly measured entities (the analysis unit is an episode of individual hospitalization; thus, it can consider the case in which the same individual is readmitted). This model assumes an appropriate distribution for each individual, considers the correlation between individuals, and handles unbalanced data with correlated results and missing values. Additionally, hospital fixed effects were included in the model to account for hospital‐specific differences. An independent subgroup analysis was performed to investigate the combined effects of hospitalization and other covariates on in‐hospital mortality. Results were reported as odds ratios [OR] and 95% confidence intervals [CI] to assess the risk of in‐hospital mortality across different groups. Statistical analyses were performed using SAS software (version 9.4; SAS Institute, Cary, NC, USA), and statistical significance was determined using a two‐tailed test with a *p*‐value set at 0.05. All analyses were performed from October 16, 2023, to January 12, 2024.

## 3 Results

The general characteristics and distribution of the study population after 1:3 matching are presented in Tables 1 and 2. Among the 398,732 patients with cancer, 205,819 male (51.6%) and 192,912 female (48.4%) inpatients were eligible for analysis. The proportion of patients with cancer in their 60s (119,260 [29.9%]) and 70s (91,872 [23.0%]), metropolitan residents (210,118 [52.7%]), and high‐income residents (141,519 [35.5%]) was high. In addition, the proportions of primary colorectal (60,784 [15.2%]), breast (50,746 [12.7%]), and lung (46,176 [11.6%]) cancers were higher than those of other cancers. Patients with cancer are mainly hospitalized in tertiary hospitals (334,699 [83.9%]), hospitals located in metropolitan areas (298.219 [74.8%]), and hospitals with a large number of beds (166,201 [41.7%]). In‐hospital mortality occurred in approximately 11,138 (2.8%) patients, while 2.8% of cancer patients who did not use hospitalist wards had in‐hospital mortality, and there was no significant difference between the groups (*p* = 0.1654).

**TABLE 1:** General characteristics of the study population.

Variables	Total		Mortality in hospital				*p*
			Yes		No		
			*N*	%	*N*	%	
Total	398,732	100.00	11,138	2.8	387,594	97.2	
Hospitalist system							
Use	99,683	25.00	2722	24.4	96,961	25.0	0.1654
None	299,049	75.00	8416	75.6	290,633	75.0	
Sex							
Male	205,819	51.62	7047	63.3	198,772	51.3	< 0.001
Female	192,913	48.38	4091	36.7	188,822	48.7	
Age							
< 30	21,692	5.44	153	1.4	21,539	5.6	< 0.001
30–39	9926	2.49	149	1.3	9777	2.5	
40–49	34,305	8.60	460	4.1	33,845	8.7	
50–59	74,514	18.69	1326	11.9	73,188	18.9	
60–69	119,260	29.91	2956	26.5	116,304	30.0	
70–79	91,872	23.04	3170	28.5	88,702	22.9	
≥ 80	47,163	11.83	2924	26.3	44,239	11.4	
Region							
Metropolitan	210,118	52.70	6073	54.5	204,045	52.6	< 0.001
Urban	68,635	17.21	1965	17.6	66,670	17.2	
Rural	119,979	30.09	3100	27.8	116,879	30.2	
Income level							
Q1 (Low)	92,190	23.12	2617	23.5	89,573	23.1	< 0.001
Q2	73,713	18.49	1906	17.1	71,807	18.5	
Q3	91,310	22.90	2461	22.1	88,849	22.9	
Q4 (High)	141,519	35.49	4154	37.3	137,365	35.4	
Hospital type							
Tertiary hospital	334,699	83.94	8365	75.1	326,334	84.2	< 0.001
General hospital	64,033	16.06	2773	24.9	61,260	15.8	
Hospital region							
Metropolitan	298,219	74.79	7518	67.5	290,701	75.0	< 0.001
Urban	38,556	9.67	1634	14.7	36,922	9.5	
Rural	61,957	15.54	1986	17.8	59,971	15.5	
Number of beds							
Q1 (low)	33,359	8.37	1653	14.8	31,706	8.2	< 0.001
Q2	115,103	28.87	3259	29.3	111,844	28.9	
Q3	84,069	21.08	2787	25.0	81,282	21.0	
Q4 (high)	166,201	41.68	3439	30.9	162,762	42.0	
Charlson Comorbidity Index							
0	347,903	87.25	8329	74.8	339,574	87.6	< 0.001
1	18,326	4.60	650	5.8	17,676	4.6	
≥ 2	32,503	8.15	2159	19.4	30,344	7.8	
Primary cancer type							
Lung cancer	46,176	11.58	2659	23.9	43,517	11.2	< 0.001
Liver cancer	23,280	5.84	1049	9.4	22,231	5.7	
Colorectal cancer	60,784	15.24	1076	9.7	59,708	15.4	
Gastric cancer	25,728	6.45	808	7.3	24,920	6.4	
Pancreatic cancer	24,121	6.05	892	8.0	23,229	6.0	
Gallbladder/bile duct cancer	12,254	3.07	493	4.4	11,761	3.0	
Breast cancer	50,746	12.73	490	4.4	50,256	13.0	
Prostate cancer	6278	1.57	138	1.2	6140	1.6	
Non‐Hodgkim's lymphoma	9958	2.50	295	2.6	9663	2.5	
Leukemia	10,460	2.62	422	3.8	10,038	2.6	
Others	128,947	32.34	2816	25.3	126,131	32.5	

**TABLE 2:** Distribution of the study population after 1:3 matching.

Variables	Total		Using hospitalist system				*p*
			Case		Control		
			*N*	%	*N*	%	
Total	398,732	100.0	99,683	25.0	299,049	75.0	
Sex							
Male	205,819	51.6	51,458	51.6	154,361	51.6	0.9810
Female	192,913	48.4	48,225	48.4	144,688	48.4	
Age							
< 30	21,692	5.4	5420	5.4	16,272	5.4	1.0000
30–39	9926	2.5	2483	2.5	7443	2.5	
40–49	34,305	8.6	8576	8.6	25,729	8.6	
50–59	74,514	18.7	18,631	18.7	55,883	18.7	
60–69	119,260	29.9	29,815	29.9	89,445	29.9	
70–79	91,872	23.0	22,968	23.0	68,904	23.0	
≥ 80	47,163	11.8	11,790	11.8	35,373	9.1	
Region							
Metropolitan	210,118	52.7	49,819	50.0	160,299	53.6	< 0.001
Urban	68,635	17.2	17,188	17.2	51,447	17.2	
Rural	119,979	30.1	32,676	32.8	87,303	29.2	
Income level							
Q1 (Low)	92,190	23.1	22,392	22.5	69,798	23.3	< 0.001
Q2	73,713	18.5	18,611	18.7	55,102	18.4	
Q3	91,310	22.9	22,863	22.9	68,447	22.9	
Q4 (High)	141,519	35.5	35,817	35.9	105,702	35.3	
Hospital type							
Tertiary hospital	334,699	83.9	83,675	83.9	251,024	83.9	1.0000
General hospital	64,033	16.1	16,008	16.1	48,025	16.1	
Hospital region							
Metropolitan	298,219	74.8	74,556	74.8	223,663	74.8	1.0000
Urban	38,556	9.7	9639	9.7	28,917	9.7	
Rural	61,957	15.5	15,488	15.5	46,469	15.5	
Number of beds							
Q1 (low)	33,359	8.4	7818	7.8	25,541	8.5	< 0.001
Q2	115,103	28.9	29,684	29.8	85,419	28.6	
Q3	84,069	21.1	14,368	14.4	69,701	23.3	
Q4 (high)	166,201	41.7	47,813	48.0	118,388	39.6	
Charlson Comorbidity Index							
0	347,903	87.3	86,505	86.8	261,398	87.4	< 0.001
1	18,326	4.6	4724	4.7	13,602	4.5	
≥ 2	32,503	8.2	8454	8.5	24,049	8.0	
Primary cancer type							
Lung cancer	46,176	11.6	11,545	11.6	34,631	11.6	1.0000
Liver cancer	23,280	5.8	5820	5.8	17,460	5.8	
Colorectal cancer	60,784	15.2	15,196	15.2	45,588	15.2	
Gastric cancer	25,728	6.5	6432	6.5	19,296	6.5	
Pancreatic cancer	24,121	6.0	6030	6.0	18,091	6.0	
Gallbladder/bile duct cancer	12,254	3.1	3064	3.1	9190	3.1	
Breast cancer	50,746	12.7	12,688	12.7	38,058	12.7	
Prostate cancer	6278	1.6	1570	1.6	4708	1.6	
Non‐Hodgkim's lymphoma	9958	2.5	2489	2.5	7469	2.5	
Leukemia	10,460	2.6	2615	2.6	7845	2.6	
Others	128,947	32.3	32,234	32.3	96,713	32.3	

**FIGURE 1:**
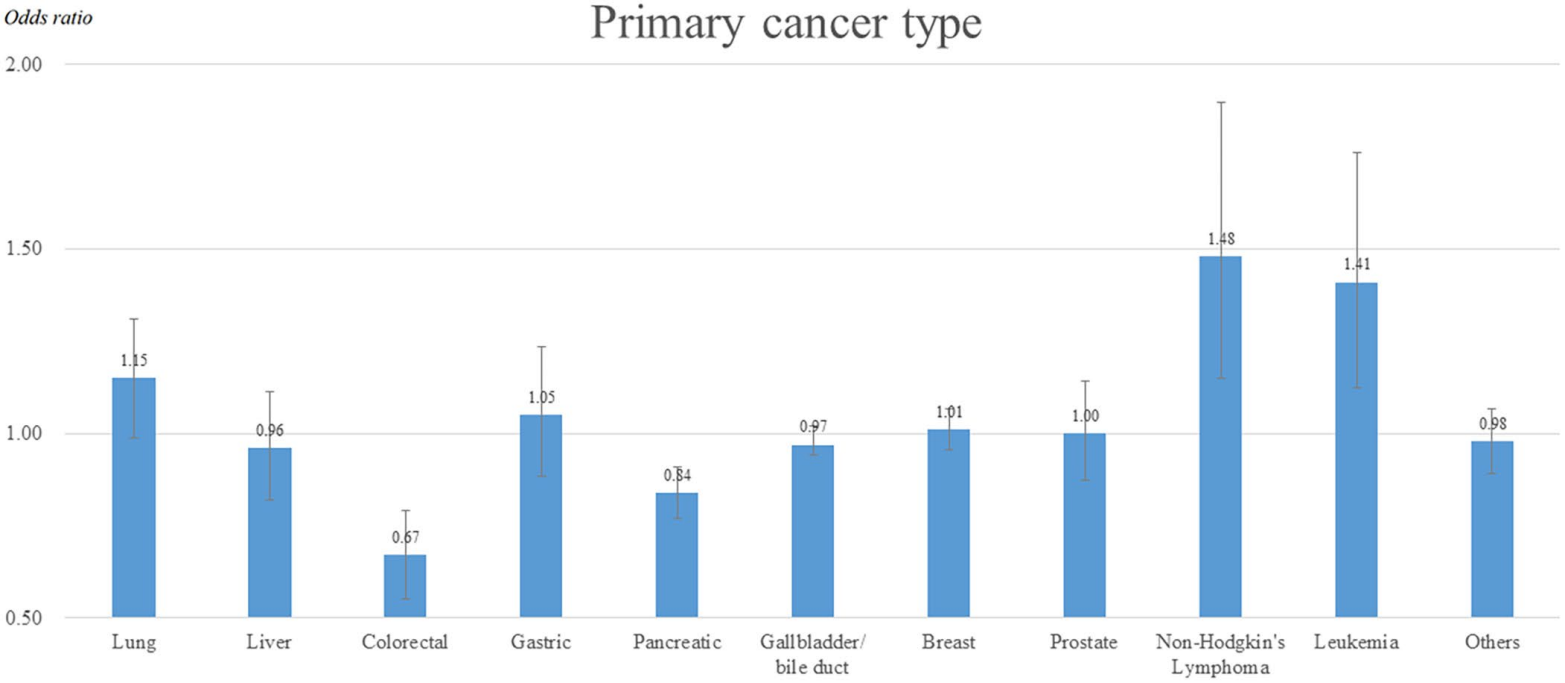
Results of subgroup analysis of risk of in‐hospital mortality by primary cancer type.

Table 3 shows the association between the use of the hospitalist system and in‐hospital mortality after adjusting for covariates. The probability of in‐hospital mortality was 9% lower in patients who used the hospitalist system than in those in the general ward (OR: 0.91; 95% CI: 0.87–0.96). Figure 1 shows the in‐hospital mortality ORs for each primary carcinoma in patients who received the hospitalist system. In‐hospital mortality according to the inpatient hospitalist system decreased significantly in patients with colorectal cancer (OR: 0.67, 95% CI: 0.55–0.79) and pancreatic cancer (OR: 0.84, 95% CI: 0.77–0.91), which were statistically significant. The risk of in‐hospital mortality was high in patients with non‐Hodgkin's lymphoma (OR: 1.48, 95% CI: 1.15–1.90) and leukemia (OR: 1.41, 95% CI: 1.12–1.76).

**TABLE 3:** The association between hospitalist system and in‐hospital mortality among cancer patients adjusted for all covariates.

Variables	Mortality in hospital	
	OR	95% CI
Hospitalist system		
Use	0.91	0.87–0.96
None	1.00	
Sex		
Male	1.28	1.22–1.34
Female	1.00	
Age		
< 30	1.00	
30–39	5.48	3.98–7.55
40–49	5.15	3.84–6.91
50–59	6.02	4.54–7.98
60–69	7.40	5.60–9.77
70–79	9.46	7.17–12.50
≥ 80	17.68	13.39–23.34
Region		
Metropolitan	1.45	1.36–1.55
Urban	1.05	0.98–1.13
Rural	1.00	
Income level		
Q1 (Low)	1.00	
Q2	0.99	0.93–1.05
Q3	0.99	0.93–1.06
Q4 (High)	0.99	0.93–1.04
Hospital type		
Tertiary hospital	1.00	
General hospital	1.33	1.23–1.45
Hospital region		
Metropolitan	1.00	
Urban	1.56	1.43–1.70
Rural	1.59	1.46–1.73
Number of beds		
Q1 (low)	1.60	1.44–1.79
Q2	1.01	0.94–1.09
Q3	1.08	1.01–1.15
Q4 (high)	1.00	
Charlson Comorbidity Index		
0	1.00	
1	1.16	1.07–1.26
≥ 2	2.52	2.34–2.71
Primary cancer type		
Lung cancer	2.23	2.10–2.37
Liver cancer	2.13	1.96–2.31
Colorectal cancer	0.73	0.68–0.79
Gastric cancer	1.33	1.22–1.46
Pancreatic cancer	1.77	1.63–1.93
Gallbladder/bile duct cancer	1.71	1.53–1.91
Breast cancer	0.63	0.56–0.71
Prostate cancer	0.76	0.63–0.91
Non‐Hodgkim's lymphoma	1.74	1.53–1.98
Leukemia	4.71	4.13–5.37
Others	1.00	

We assumed that the quality of healthcare would vary according to personal characteristics; therefore, we performed subgroup analyses stratified by age and CCI scores. As shown in Table 4, older adults under the hospitalist system had a lower risk of in‐hospital mortality (70–79 years, OR: 0.82, 95% CI: 0.74–0.89; ≥ 80 years, OR: 0.31, 95% CI: 0.19–0.52). In addition, the lowest risk of in‐hospital mortality was found in patients with comorbidities (CCI score ≥ 2, OR: 0.93, 95% CI: 0.87–0.99).

**TABLE 4:** Results of subgroup analysis stratified by age and Charlson Comorbidity Index.

Variables	Mortality in hospital		
	None	Use hospitalist system	
	OR	OR	95% CI
Age			
< 30	1.00	1.32	0.98–1.61
30–39	1.00	1.21	0.98–1.44
40–49	1.00	1.23	0.94–1.52
50–59	1.00	1.09	0.99–1.18
60–69	1.00	0.99	0.91–1.07
70–79	1.00	0.82	0.74–0.89
≥ 80	1.00	0.31	0.19–0.52
Charlson Comorbidity Index			
0	1.00	1.20	1.09–1.33
1	1.00	0.96	0.91–1.00
≥ 2	1.00	0.93	0.87–0.99

## 4 Discussion

In Korea, there is an ongoing discussion about the shortage of medical personnel compared to the number of hospital beds. Various hospitalist services caring for inpatients throughout their stay have been institutionally established. The hospitalist system for inpatient care was initiated as a pilot project in 2015 and expanded to the main project in 2021, with a recent government policy aimed at improving the quality of inpatient health care. Specifically, the placement of hospitalists in intensive care units of tertiary hospitals is currently mandatory; however, that for inpatients is not. Studies on the effectiveness of the hospitalist system in Korea are lacking despite policy introductions. Despite advancements in diagnostic and treatment technologies, leading to improved survival rates among patients with cancer, cancer‐related hospitalizations have been increasing.

In this retrospective cohort study, we investigated whether the quality of healthcare for hospitalized patients with cancer in Korea has improved. The main findings of this study were as follows: In‐hospital mortality in patients using the inpatient hospitalist system decreased by 9%, which was noticeable in older adults and patients with many comorbidities. In addition, the introduction of the hospitalist system for patients with primary carcinoma, such as colorectal and pancreatic cancer, contributed to a reduction in hospital deaths. Hospitalists are responsible for managing patients from admission to discharge, providing a more continuous and accountable care model than in other countries where there may be designated primary care physicians or where care is fragmented. This responsibility likely improves patient outcomes by ensuring better coordination and more consistent management throughout the hospital stay. We hypothesize that hospitalists reduce mortality through several key mechanisms: continuous care, better patient monitoring, and rapid decision‐making. The presence of hospitalists may allow for quicker response times and more efficient treatment plans, which are crucial in managing complex patients and preventing deterioration. This ongoing care is especially important in cases where patients' conditions fluctuate, as hospitalists can immediately address complications or make timely adjustments to treatment plans. In Korea, non‐hospitalist care for oncology patients is typically managed by specialist oncologists or general internists, with varying care structures. Non‐hospitalist teams often consist of attending physicians, residents, and allied health professionals (APPs), with care being less continuous compared to the hospitalist model, where a single physician manages the patient from admission to discharge. These issues underscore the importance of considering both the clinical expertise of hospitalists and the potential need for specialized co‐management, particularly in complex cancer cases. Future studies should examine how hospitalist care for different cancer types may be optimized and how collaborations with specialists in various cancer subtypes could improve patient outcomes.

Most previous studies evaluating inpatient hospitalists have focused on parameters such as lengths of stay (LOS), readmissions, hospital‐acquired infections, and costs. These studies have consistently shown a decrease in these outcomes when inpatients under the hospitalist system were involved [16, 27, 29, 30]. One study reported that older adult patients under a geriatrician hospitalist system experienced shorter LOS and reduced readmissions compared to general patients [16]. A large‐scale RAND study suggested the efficiency of geriatric specialists in the inpatient care of older adults, citing shortened hospital stays and cost savings per admission as positive outcomes [29]. Our results support the findings of previous studies that in‐hospital mortality decreases when older adult patients receive hospitalist services compared to when they do not. Similar to several previous studies indicating a decrease in LOS in patients with multiple comorbidities [27, 30], our study found a reduction in in‐hospital mortality in patients with high CCI scores, suggesting that the inpatient hospitalist system improved the quality of healthcare for patients with severe or coexisting conditions by providing meticulous management when necessary. Our findings support the broader understanding that hospitalist care improves patient outcomes, not just in terms of mortality but also in terms of improved coordination of care and reduced length of stay. Especially, hospitalists have demonstrated a reduction in inpatient mortality, particularly in the co‐management of surgical patients, where their involvement in post‐operative care has been shown to improve outcomes through better continuity of care and faster decision‐making [31, 32]. This has been observed across various specialties, such as in geriatric care, where hospitalists have contributed to shorter lengths of stay and reduced readmissions, which indirectly contribute to improved survival outcomes [33].

We found that in‐hospital mortality was significantly lower in patients with colorectal and pancreatic cancers and higher in patients with non‐Hodgkin's lymphoma and leukemia. This finding suggests that hospitalists may have different levels of effectiveness in treating various cancer types. Specifically, inpatient hospitalists, owing to their potentially limited clinical experience compared with hospitalists specializing in specific cancer subtypes, may face challenges in treating more diverse and rare forms of cancer. This could result in a slower response to treatment or less effective management for cancers that require highly specialized care [23]. In Korea, hospitalists typically specialize in family medicine, general surgery, or similar fields. There are no additional requirements for hospitalists to pursue further specialization in oncology or hematology. Therefore, while hospitalists in Korea may be well‐versed in managing common cancers, such as colorectal cancer, which have higher prevalence rates, they may lack the specialized knowledge needed to treat less common hematologic malignancies like lymphoma and leukemia. This could explain the higher mortality observed in these patients under hospitalist care.

These issues underscore the importance of considering both the clinical expertise of hospitalists and the potential need for specialized co‐management, particularly in complex cancer cases. Future studies should examine how hospitalist care for different cancer types may be optimized, and how collaborations with specialists in various cancer subtypes could improve patient outcomes. Also, in line with studies reporting that management through the inpatient hospitalist system after surgery can effectively manage medical complications and facilitate efficient communication [34], future studies should consider factors such as cancer stage, metastatic status, and whether surgery was performed. However, while factors such as cancer stage, metastatic status, and whether surgery was performed during the hospital stay may provide valuable information, they may have a limited impact on determining how best to structure care in inpatient units. For example, we would not necessarily change the management of metastatic cancer patients based solely on their disease stage. Therefore, our study underscores the importance of understanding patient outcomes based on the type of physician (hospitalists, oncology‐specific hospitalists, and general physician) managing the patients. This distinction is vital for shaping healthcare strategies and understanding the real‐world impact of different care models on cancer patients.

This study has some limitations. First, the analyzed NHIS cohort data did not include information on the stage of the cancer. Therefore, we conducted a detailed analysis by categorizing the major cancer types with high mortality rates into 10 groups. Future research on specific cancer types should be conducted in consideration of the stage of cancer. Additionally, while we excluded patients admitted to hospice wards to minimize the inclusion of terminal cancer patients, not all terminal cancer patients may necessarily be in hospice wards. Therefore, the mortality rate observed could potentially be influenced by terminal cancer patients who were not admitted to hospice care wards. Furthermore, the dataset's limitations also hinder the differentiation between cancer‐related admissions and those for chemotherapy or immunotherapy. Future studies should aim to better classify cancer patients based on admission type and disease stage, which would provide more accurate insights into the outcomes of hospitalist care. Additionally, the findings are specific to cancer patients, and the generalizability of the hospitalist model's effects to other populations is uncertain. Further studies are necessary to determine whether the benefits of hospitalist care extend to patients with other conditions or diseases. Second, there were inherent limitations to the administrative claims data. The NHIS cohort dataset is not a mandatory record but is primarily collected for the purpose of reimbursing insurers for part of the total treatment costs. Therefore, the disease codes recorded in the NHIS may not fully reflect the actual medical conditions of the patients. Third, despite the use of ICD‐10 codes for participant selection and specific outcome verification, these codes have certain constraints. The ICD‐10 codes are primarily used for administrative purposes and may not provide detailed clinical information about patients. Thus, there is a possibility of incomplete coding, leading to misclassification or underestimation of the results. However, to enhance diagnostic accuracy, we included all secondary diagnosis codes in our analyses. Fourth, in South Korea, inpatient hospitalist systems are only implemented in a few tertiary and general hospitals. Therefore, there is a possibility of upward standardization when comparing the quality of medical care with that of other hospitals, and generalizing the results to outpatient hospitals may be challenging. Fifth, this study focused on in‐hospital mortality as the primary outcome to assess the effect of hospitalists stationed in the hospital. However, in future research, it will be important to consider additional mortality outcomes, such as 30‐day and 90‐day mortality, in order to provide a more comprehensive understanding. Sixth, examining the characteristics of these groups before matching would have been valuable; it was not feasible within the constraints of our data. Finally, we could not completely exclude potential and residual confounding factors due to unmeasured variables.

In conclusion, the establishment of inpatient hospitalist systems reduces in‐hospital mortality among patients with cancer, indicating their potential contribution to improving the quality of healthcare in Korea. However, the association observed between hospitalist services and reduced in‐hospital mortality is specific to cancer patients, and its generalizability to other patient populations remains unclear. Further research is needed to explore the effects of hospitalist care across different patient groups. Our results emphasize the need for dedicated personnel stationed in hospital wards to improve short‐ and long‐term survival outcomes of patients with cancer, with particular emphasis on prioritizing deployment for older patients and those with multiple comorbidities. Furthermore, the government should consider mandatory expansion of the current policy and alignment with the characteristics of hospitals in Korea to contribute to the efficient allocation of healthcare resources.

## Author Contributions

**Yun Seo Jang:** conceptualization (equal), data curation (lead), formal analysis (lead), investigation (equal), methodology (equal), visualization (lead), writing – original draft (lead). **Il Yun:** data curation (supporting), formal analysis (supporting), methodology (equal), resources (lead), writing – review and editing (supporting). **Yu Shin Park:** conceptualization (equal), formal analysis (supporting), investigation (supporting), methodology (supporting), validation (equal). **Eun‐Cheol Park:** methodology (supporting), supervision (equal), validation (equal), visualization (supporting), writing – review and editing (supporting). **Jaeyong Shin:** methodology (equal), supervision (lead), validation (equal), visualization (supporting), writing – review and editing (lead).

## Disclosure

Precis: The inpatient hospitalist system improves the quality of healthcare by reducing the in‐hospital mortality rate of cancer patients. Especially, for cancer patients with old age or multiple comorbidities, we highlight the need for dedicated impatient hospitalist in wards.

## Conflicts of Interest

The authors declare no conflicts of interest.

## Supporting information


**Appendix S1:** Status of hospitalist operating institutions in Korea.

## Data Availability

The Korean National Health Insurance Sharing Service (NHISS) operates a publicly accessible website for data sets, which can be found at https://nhiss.nhis.or.kr/ and is managed by the Korean NHIS.
