# Correction to “Implementation of the Hospitalist System and In‐Hospital Mortality Among Patients With Cancer: Using the National Health Insurance Cohort Data”

**DOI:** 10.1002/cam4.71479

**Published:** 2025-12-17

Jang YS, Yun I, Park YS, Park EC, Shin J. Implementation of the Hospitalist System and In‐Hospital Mortality Among Patients With Cancer: Using the National Health Insurance Cohort Data. *Cancer Medicine*, 2025;*14*(19), e71207.

The “Funding” section on p. 1 was incorrect in the originally published version of the article. The text “The authors received no specific funding for this work” should read as follows: “This research was supported by the Ministry of Trade, Industry & Energy (MOTIE) (project no. RS‐2024‐00432987).”

We apologize for this error.

