# Supplementary material for: Implementation of the Hospitalist System and In‐Hospital Mortality Among Patients With Cancer: Using the National Health Insurance Cohort Data
Source: Cancer Med. 2025 Oct 2;14(19):e71207. doi: 10.1002/cam4.71207 (PMC12491651; doi:10.1002/cam4.71207)
Supplement: Supplementary file 1 — Appendix S1: Status of hospitalist operating institutions in Korea. [file CAM4-14-e71207-s001.docx]

| **Supplementary 1. Status of Hospitalist operating institutions in Korea** | | |
| --- | --- | --- |
|  | 2021 | 2022 |
| Number of hospital | 48 | 56 |
| Number of ward | 147 | 162 |
| Number of hospitalist | 276 | 303 |
| hospitalist per 1 ward | 1.88 | 1.87 |
